# Extra-mitochondrial citrate synthase initiates calcium oscillation and suppresses age-dependent sperm dysfunction

**DOI:** 10.1038/s41374-019-0353-3

**Published:** 2019-12-19

**Authors:** Woojin Kang, Yuichirou Harada, Kenji Yamatoya, Natsuko Kawano, Seiya Kanai, Yoshitaka Miyamoto, Akihiro Nakamura, Mami Miyado, Yoshiki Hayashi, Yoko Kuroki, Hidekazu Saito, Yasuhiro Iwao, Akihiro Umezawa, Kenji Miyado

**Affiliations:** 1 Department of Reproductive Biology, National Research Institute for Child Health and Development, 2-10-1 Okura, Setagaya, Tokyo, 157-8535 Japan; 2 Department of Perinatal Medicine and Maternal Care, National Center for Child Health and Development, 2-10-1 Okura, Setagaya, Tokyo, 157-8535 Japan; 3 Institute for Environmental and Gender-Specific Medicine, Juntendo University Graduate School of Medicine, 2-1-1 Tomioka, Urayasu-City, Chiba 279-0021 Japan; 4 Department of Life Sciences, School of Agriculture, Meiji University, 1-1-1 Higashi-Mita, Tama-ku, Kawasaki-shi, Kanagawa 214-8571 Japan; 5 Department of Molecular Endocrinology, National Research Institute for Child Health and Development, 2-10-1 Okura, Setagaya, Tokyo, 157-8535 Japan; 6 Life Science Center for Survival Dynamics, Tsukuba Advanced Research Alliance (TARA), University of Tsukuba, 1-1-1 Tennodai, Tsukuba, Ibaraki 305-8572 Japan; 7 Department of Genome Medicine, National Research Institute for Child Health and Development, 2-10-1 Okura, Setagaya, Tokyo, 157-8535 Japan; 8 Division of Earth Science, Biology, and Chemistry, Graduate School of Sciences and Technology for Innovation, Yamaguchi University, 1677-1 Yoshida, Yamaguchi City, Yamaguchi 753-8511 Japan; 9 Present Address: Department of Molecular Pathology, Tokyo Medical University, 6-1-1 Shinjuku, Shinjuku, Tokyo 160-8402 Japan

**Keywords:** Ageing, Reproductive biology

## Abstract

Men and women become infertile with age, but the mechanism of declining male fertility, more specifically, the decrease in in sperm quality, is not well known. Citrate synthase (CS) is a core enzyme of the mitochondrial tricarboxylic acid (TCA) cycle, which directly controls cellular function. Extra-mitochondrial CS (eCS) is produced and abundant in the sperm head; however, its role in male fertility is unknown. We investigated the role of eCS in male fertility by producing *eCs*-deficient (*eCs*-KO) mice. The initiation of the first spike of Ca^2+^ oscillation was substantially delayed in egg fused with *eCs*-KO sperm, despite normal expression of sperm factor phospholipase C zeta 1. The *eCs*-KO male mice were initially fertile, but the fertility dropped with age. Metabolomic analysis of aged sperm revealed that the loss of eCS enhances TCA cycle in the mitochondria with age, presumably leading to depletion of extra-mitochondrial citrate. The data suggest that eCS suppresses age-dependent male infertility, providing insights into the decline of male fertility with age.

## Abstract (web summary)

Citrate synthase is a core enzyme of tricarboxylic acid cycle, but the role of extra-mitochondrial CS (eCS) is less clear. eCS, abundant in the sperm head, triggers a first spike of egg Ca^2+^ oscillation in a phospholipase C zeta 1 -independent manner. Moreover, the fertility of eCs-knockout male mice dropped with age, indicating that eCS suppresses age-dependent sperm dysfunction by accelerating Ca^2+^ oscillation.

## Introduction

Age affects male fertility indirectly and directly. Female fertility declines after the age of 35, thus indirectly affecting and often diminishing the male fertility [1]. More directly, a decline in male infertility is observed as a rapid decrease of sperm quality and more severe problems that lead to azoospermia [2]. A number of factors, including age, reduce one’s ability to produce sperm, ejaculate, and sperm quality, all of which are required for successful fertilization [3]. However, the causes of the decline of male fertility with age are multiple and still undefined.

**Fig. 1:**
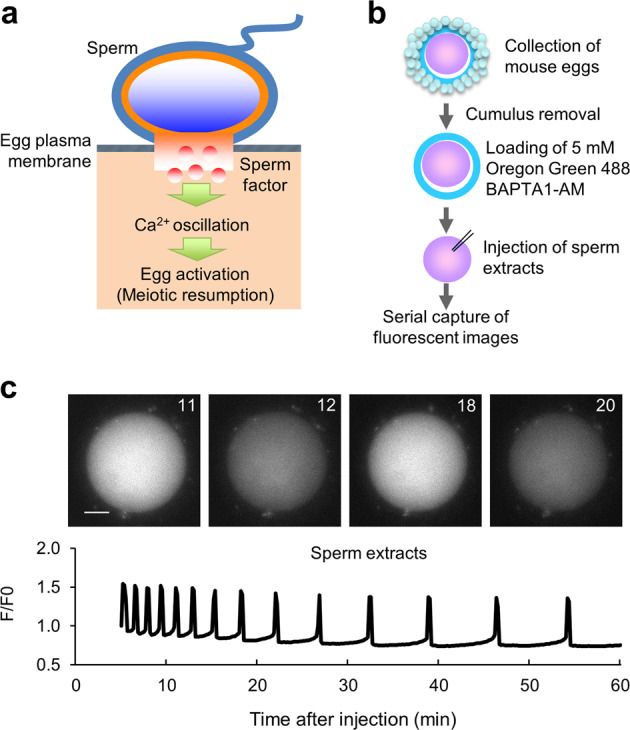
Ca^2+^ oscillation induced by injection of the sperm extract. **a** Schematic overview of Ca^2+^ oscillation induced by the sperm factor. **b** Experimental flow for the measurement of increases of Ca^2+^ levels in the egg. **c** Ca^2+^ oscillation after the injection of mouse sperm extracts. Mouse sperm extracts were prepared from sperm of 8- to 12-week-old 15 male B6C3F1 mice (three male mice per experiment). The pattern of Ca^2+^ oscillation in eggs injected with Oregon green after the injection of sperm extracts (0.2 pg/egg). Sperm extracts were injected into the eggs at 0 min, and the fluorescent images were then acquired every 10 s. A total of 42 eggs were examined in quintuplicate experiments. The ratio (*F*/*F*_0_) of the fluorescence intensity before the injection (*F*_0_) to that after the injection (*F*) is shown.

Upon cell stimulation by external stimuli, calcium ions (Ca^2+^) are released from the intracellular stores, such as the endoplasmic reticulum [4]. This results in an oscillatory or transient increase in intracellular Ca^2+^ concentration [5]. Hence, Ca^2+^ is categorized as a secondary messenger of the transduction of external signals into cellular events [6]. After the sperm and egg fuse, the mammalian egg proceeds to meiotic metaphase II (MII), termed egg activation [7], a process that is initiated by Ca^2+^ oscillation (Fig. 1a) [8].

Phospholipase C zeta 1 (PLCz1) is a sperm factor that triggers Ca^2+^ oscillation in the egg [8]. Oscillation is induced in mouse and human eggs after the microinjection of exogenous *Plcz1* poly(A)^+^-RNA or microinjection of a recombinant PLCz1 protein [9]. In both cases, the patterns of Ca^2+^ oscillation are similar to those induced by a sperm extract (Fig. 1b, c) and those following in vitro fertilization (IVF) [8]. Male mice lacking *Plcz1* are subfertile [10, 11], indicating that other mechanisms are involved in egg activation.

There is an intriguing idea concerning Ca^2+^ oscillation during mammalian egg activation [12]. It has been suggested that Ca^2+^ oscillations occur in two major stages, a primary spike in Ca^2+^ levels, followed by repetitive spikes of Ca^2+^ levels that last several hours. While the source of the first Ca^2+^ spike is the endoplasmic reticulum of the egg, the ensuing repetitive Ca^2+^ spikes cease in the absence of extracellular Ca^2+^, implying that the two stages are regulated by intracellular and extracellular Ca^2+^ sources, respectively. The repetitive changes in Ca^2+^ levels are essential for the mammalian egg activation. The induction of the first Ca^2+^ spike is likewise important for the mammalian egg activation. Indeed, a transient increase in Ca^2+^ levels suffices to trigger egg activation in several animal species [13].

The newt *Cynops pyrrhogaster* exhibits physiological polyspermic fertilization, during which multiple sperm enter a single egg [14]. Harada et al. [15] discovered that microinjection of a mitochondrial citrate synthase (CS) into unfertilized eggs induces a transient intracellular calcium ion concentration ([Ca^2+^]_i_) rise. CS is localized in the mitochondrial matrix, where it catalyzes the formation of citric acid from acetyl-coenzyme A (CoA) and oxaloacetate [16]. In addition, palmitoyl-CoA, an inhibitor of CS, prevents egg activation, and the injection of acetyl-CoA or oxaloacetate promotes egg activation [16], suggesting wide function of CS during male reproduction in the vertebrates.

Otherwise, Ca^2+^ signaling regulates several sperm functions including sperm capacitation, motility, and hyperactivation [17]. Ca^2+^ storage in the sperm is also involved in sperm penetration into the extracellular matrix covering an egg, zona pellucida (ZP), after removal of the sperm outer membrane, acrosome reaction (AR) [18]. It is assumed that intercellular and intracellular Ca^2+^ dynamics are absolutely essential for sperm function because of its direct effect on sperm metabolism.

Metabolomic analysis is useful for measurement of metabolites present in various samples including cells, tissues, and fluids [19, 20]. Recent studies revealed metabolomic profiles in aging models and identified candidates of age-related biomarkers in human cells and tissues [21, 22]. For example, reactive oxygen species (ROS)-related factors, and spontaneously generated superoxide, which have a negative effect on sperm motility [23]. Furthermore, ROS is one of causal substances in patients with azoospermia [24]. Correspondingly, rat testes exhibit a remarkably age-dependent alteration of metabolites [25].

In various species of plants and animals, a single gene encodes protein variants corresponding to CS and extra-mitochondrial CS (eCS) (referred to as “CS-like” in the NCBI database). Hence, deletion of the *Cs* gene inhibits cellular respiration in all types of cells, making it impossible to examine the role of eCS. However, a second *Cs* gene (known as *Cs-like*, and *Csl*) (hereafter referred to *eCs*) encodes a putative eCS in mice.

Considering these previous findings [12, 16], we hypothesized that eCS may contribute to Ca^2+^ oscillation in the mouse egg, more specifically, to the induction of the first Ca^2+^ level increase. We tested this hypothesis using a novel mouse lacking the *eCs* gene (the *eCs*-KO mouse model).

## Materials and methods

### Animals

All mice were housed (5 mice or less/cage) under specific pathogen-free controlled conditions. Food and water were available ad libitum. All animal experiments were approved by the Institutional Animal Care and Use Committee of the National Research Institute for Child Health and Development (experimental number A2004-004).

For all experiments, wild-type (WT) (8–12-week-old), *eCs*-KO (8–12-week-old), WT (6-month-old), and *eCs*-KO (6-month-old) male mice were randomly divided into control and experimental groups. Each data was obtained from five to six male mice per genotype. In mating experiment for in vivo fertility, nine WT and *eCs*-KO (8–12-week-old) male mice were tested and ten older WT and 20 *eCs*-KO (6-month-old) male mice were mated.

### Antibodies and reagents

For immunoblotting and immunocytochemistry experiments, a rabbit polyclonal antibody (polyAb) against a synthetic peptide corresponding to the N-terminus (IYRNLYREDRNIEA) of the mouse eCS (GenBank accession no. NP_082221.2, 1/250 dilution for immunoblotting, 1/100 dilution for immunostaining) was produced by Japan Lamb Ltd (Hiroshima, Japan). Rabbit anti-CS polyAb (NE040/7S, 1/500 dilution for immunoblotting, 1/100 dilution for immunostaining) and anti-PLCz1 polyAb (ab181816, 1/250 dilution for immunoblotting, 1/50 dilution for immunostaining) were purchased from Nordic MUbio (Susteren, Netherlands) and Abcam Inc. (Cambridge, MA), respectively. Mouse anti-β-actin mAb (AC-15, 1/500 dilution for immunoblotting) and anti-glyceraldehyde-3-phosphate dehydrogenase mAb (5A12, 1/500 dilution for immunoblotting) were purchased from Sigma-Aldrich (St. Louis, MO) and WAKO Pure Chemical Industries (Osaka, Japan). IgGs conjugated with Alexa Fluor 488 and 546 were purchased from Molecular Probes (Invitrogen, Carlsbad, CA) as secondary antibodies for immunohistochemistry. Horseradish peroxidase-conjugated secondary Abs (Sigma-Aldrich) were used for immunoblotting. The nuclei were counterstained with 4′,6-diamidino-2-phenylindole (WAKO Pure Chemical Industries, Osaka, Japan).

### Collection of ovulated eggs

Female BDF1 mice (8–12-week old; purchased from Japan SLC Inc., Shizuoka, Japan) received intraperitoneal injections of 5 IU (100 μl) of the pregnant mare’s serum gonadotropin (Merck4Biosciences, Darmstadt, Germany) followed by 5 IU (100 μl) of human chorionic gonadotropin (hCG; Merck4Biosciences) 48 h later. MII-stage eggs were collected from the oviducts of females 14–16 h after the administration of hCG. The cumulus cells were dispersed from the eggs by incubating for 10 min at 37 °C in TYH medium containing hyaluronidase (300 μg/ml; Merck4Biosciences). The eggs were then incubated in TYH medium at 37 °C under 5% CO_2_ in air.

### Synthesis of poly(A)^+^-RNAs

The open reading frames (ORFs) encoding mouse *Cs* and *eCs* (GenBank accession no. NM_026444.4 and NP_027945.3) cDNA were amplified by polymerase chain reaction (PCR) using the primer sets: 5′-AGCTGTAGCTCTCTCCCTTC-3′ (forward; 20-mer) and 5′-TGCTTCAGTCCCGGTCATCCTA-3′ (reverse; 22-mer) for *Cs*; and 5′-TCAGCCTTTCAACATCACTG-3′ (forward; 20-mer) and 5′-CCTCGTTTTCTTATGGGCAG-3′ (reverse; 20-mer) for *eCs*. The amplified ORFs were inserted into the pGEM-T Easy vector (Promega, Madison, WI). RNA was synthesized using the T7 MEGAScript kit (Thermo Fisher Scientific, Waltham, MA). Transcription was performed in the presence of 3′-O-Me-m7G(5′)ppp(5′)G RNA cap structure analog (New England Biolabs, Beverly, MA), using the constructed plasmids harboring *Cs* and *eCs* as templates. The synthesized RNAs were detected by agarose gel electrophoresis and their concentration quantified by measuring UV absorbance using spectrophotometer, they were then purified by chloroform treatment and precipitated with ethanol. Before injection into the mouse eggs, RNA precipitates were resuspended in a solution of 1 mM Tris-HCl (pH 8.0) and 0.1 mM EDTA. For the injection, the final concentration of RNA was adjusted to 1 μg/μl.

### Reverse-transcription PCR

To detect the *Cs* and *eCs* mRNA, total cellular RNA was prepared from mouse tissues using Isogen (Nippon Gene, Tokyo, Japan), following the manufacturer’s instructions. First-strand cDNA was synthesized from total RNA (5 μg) using Superscript III First-strand Synthesis System (Invitrogen, Carlsbad, CA). PCR was performed in a 25-μl reaction mixture containing 0.5 mg/ml cDNA and 0.5 mM each of the following primers: for *Cs*: forward, 5′-AGCTGTAGCTCTCTCCCTTC-3′, and reverse, 5′-TGCTTCAGTCCCGGTCATCCTA-3′; and for *eCS*: forward, 5′-GTTCGCCTGCCATGGCTCTACTTA-3′, and reverse, 5′-CCCCGTCTCCCATTTTATCCTGACT-3′. PCR amplification to detect *Cs* and *eCs* transcripts was performed over the course of 25 cycles of 10 s at 98 °C, 30 s each at 58 °C for *Cs* and 63 °C for *eCs*, and 1 min at 72 °C.

### RNA microinjection

The synthesized RNA was injected into the egg cytoplasm by using glass capillaries attached to a micromanipulator (Narishige Scientific Instrument Lab., Tokyo, Japan). The injection volume was 5 pl per egg, which corresponded to 2–3 pg of RNA per egg. After the injection, the Ca^2+^ oscillation patterns were observed using a fluorescence microscope (IX71, Olympus, Tokyo, Japan) with a spinning disk unit (CSU-X1, Yokogawa Electric Corporation, Tokyo, Japan).

### Immunoblotting

Mouse organs (the heart, muscle, kidney, liver, and epididymis) were collected from 8–12-week-old ICR male mice and the sperm were collected from the epididymis. The tissues were lysed by placing in Laemmli’s sodium dodecyl sulfate (SDS) sample buffer [2% (w/v) SDS, 62.5 mM Tris-HCl (pH 6.8), 0.005% bromophenol blue, and 7% glycerol] and boiling for 10 min at 95 °C. The protein samples were resolved by SDS polyacrylamide gel electrophoresis on 12% acrylamide gel, and immunoblotted. Detection of the proteins and the relevant primary Abs (0.1 μg/ml) was based on enzyme-linked color development with horseradish peroxidase-conjugated secondary Abs (0.01 μg/ml; Sigma-Aldrich).

### Immunostaining

The sperm were collected from the epididymis of 8–12-week-old ICR male mice, and fixed with 4% paraformaldehyde in HEPES-buffered saline for 1 h at 4 °C. The sperm were then incubated in TYH medium containing primary Abs (0.5 μg/ml) for 1 h at 4 °C. The sperm were next treated with secondary Abs (0.25 μg/ml; Alexa488- or Alexa546-conjugated IgG) for 1 h at 4 °C, and then washed three times with TYH medium, prior to the acquisition of fluorescent images using a laser scanning confocal microscope (LSM 510 model; Carl Zeiss Microimaging Inc., Thornwood, NY, USA). In some experiments, the sperm were visualized by immunostaining with Alexa Fluor 488-conjugated peanut lectin PNA (3 μg/ml; Molecular Probes, Invitrogen, Carlsbad, CA) on ice for 1 h, and washing with PBS. Fluorescent images were then acquired using a laser scanning confocal microscope (LSM 510 model; Carl Zeiss Microimaging Inc., Thornwood, NY).

### Generation of *eCs*-KO mouse

Mutant mice were generated from C57BL/6-derived embryonic stem cell clones by injection into blastocysts from C57BL/6 mouse with a genetically deleted *Csl* (*eCs*) (Csl^tm1(KOMP)Vlcg^; ID14519) obtained from the Knockout Mouse Project (KOMP) repository (an NCRR-NIH–supported strain suppository; www.komp.org). Homozygous mice (C57BL/6 genetic background) were generated by subsequent intercrosses of heterozygous animals.

### Electron microscopy

The sperm were collected from the epididymis of 8–12-week-old male mice and fixed in 50 mM sodium cacodylate, pH 7.2, containing 2.5% glutaraldehyde and 2% sucrose, for 2 h at 4 °C, as described previously [26]. The fixed sperm were then rinsed three times and postfixed in a fixative containing 2% osmium tetroxide for 2 h at 4 °C. The specimens were subsequently dehydrated in ethanol, immersed twice in propylene oxide for 15 min, and then embedded in Epon. Ultrathin sections were prepared by using an ultramicrotome (Reichert Ultracut; Leica AG, Vienna, Austria), and stained with uranyl acetate and lead citrate. The sections were observed using a transmission electron microscope (H-7000; Hitachi High-Tech, Tokyo, Japan).

### Measurement of sperm motility

To measure sperm motility, computer assisted sperm analysis operated by IVOS software (Hamilton-Thorne Biosciences) was used as previously described [27]. An aliquot of the capacitated sperm suspension was transferred to a prewarmed counting chamber (depth, 20 μm), and >200 sperm were examined from each sample using the standard setting (30 frames acquired at a frame rate of 60 Hz, at 37 °C). The motility of hyperactivated sperm was determined using the SORT function of the IVOS software. Sperm were classified as hyperactivated when the trajectory met the following criteria: curvilinear velocity ≥180 μm/s, linearity ≤38%, and amplitude of lateral head displacement ≥9.5 μm.

### In vitro fertilization

The eggs were collected from the oviductal ampulla region of superovulated C57BL/6N and *eCs*-KO females (8–12-week old) 14–16 h after hCG injection, and placed in a 100-μl drop of TYH medium under paraffin oil (Nacalai Tesque, Inc., Kyoto, Japan), equilibrated with 5% CO_2_ in air at 37 °C. The sperm collected from the epididymides of C57BL/6N and *eCs*-KO males (8–12-week old and 6-month old, respectively) were induced to capacitate by incubating in TYH medium for 90 min under an atmosphere of 5% CO_2_ in air at 37 °C. The eggs were then inseminated with sperm (150 sperm/μl) and two-cell embryos were observed under an Olympus IX71 microscope (Olympus) after a 24-h incubation in TYH medium at 37 °C under 5% CO_2_ in air. The 60, 122, 78, and 68 eggs were inseminated with WT, *eCs*-KO, WT (from 6-month-old males), and *eCs*-KO sperm (from 6-month-old males), respectively. These experiments were performed in quintuplicate experiments.

### Measurement of CS activity

The cauda epididymal sperm (from 8–12-week-old males) were collected in phosphate-buffered saline and washed with phosphate-buffered saline by centrifugation at 600 × *g* for 10 min. CS activity was determined using the CS activity colorimetric assay kit (Biovision, Milpitas, CA) according to the protocol provided by the manufacturer. The sperm (2 × 10^6^) were homogenized in CS buffer and incubated on ice for 10 min. After centrifugation at 10,000 × *g* for 5 min, enzyme activity was measured in the collected supernatant. The rate of color development was measured at 405 nm using a spectrophotofluorometer (Wallac ARVO^TM^ SX 1420 multilabel counter, Perkin-Elmer, Waltham, MA).

### Measurement of intracellular Ca^2+^ concentration

To monitor the changes in intracellular Ca^2+^ concentration, eggs with intact ZP (28 and 30 eggs inseminated with WT and *eCs*-KO sperm, respectively) were incubated in TYH medium containing the Ca^2+^-sensitive fluorescent dye Oregon green 488 BAPTA-1 AM (final concentration of 2 μM, Molecular Probes, Invitrogen, Carlsbad, CA) for 15 min at 37 °C in a CO_2_ incubator. They were then washed three times with TYH medium (5 min each). After washing, capacitated sperm (150 sperm/μl) were added to TYH medium. Fluorescent images of the eggs were captured using a highly sensitive CCD camera (Andor Technology, Belfast, UK), using software to operate the camera (Yokogawa, Tokyo, Japan), every 10 s. Fluorescence intensity was measured by using the imaging software Andor IQ (Andor Technology). Each fluorescent image (*F*) was subtracted from the image before injection or the image with the lowest fluorescence intensity (*F*_0_). The fluorescence intensity for individual eggs was measured within a user-selected region that covered the majority of the area of each egg. The mean intensity over the same area for each of the images in a time series was analyzed automatically. Changes in fluorescence intensity are reported as the *F*/*F*_0_ ratio after insemination with the sperm, as previously described [15].

### Metabolic analysis of the sperm

To prevent contamination of the epididymal fluids, epididymal sperm were washed with Hank’s balanced salt solution (Thermo Scientific, Rockford, IL) and immediately frozen in liquid nitrogen. Each sample (2 × 10^7^ sperm, ~20 mg) was placed in methanol (75–80% final concentration) and homogenized using zirconia beads. The homogenates were centrifuged at 18,000 × *g* for 5 min, and the supernatants were then purified on pretreated spin columns (MonoSpin C18; GL Sciences, Saitama, Japan).

The samples (each sample obtained from 2–3 male mice) were analyzed using an Agilent 7890A gas chromatography (GC) system coupled to Agilent 5975C inert XL MSD with triple-axis mass detector, an Agilent 7693 Series Autosampler, and a DB-5 capillary column (30 m × 0.25 mm i.d. × 0.25 μm film thickness; Agilent Technologies, Santa Clara, CA). Then, 0.5 μl of derivatized mixtures was injected into the inlet heated at 280 °C. A standard septum purge was performed after sample injection at 1.1 ml/min, and helium gas was used as a carrier. The transfer line, ion source, and the quadrupole were heated at 280, 200, and 150 °C, respectively. The oven was programmed initially at 100 °C for 4 min, and then ramped up to 320 °C at 8 °C/min. Ionization was performed in an electron impact mode, and the masses were scanned in the full spectrum mode from 45 to 600 m/z at 2000 u/s.

### Mitochondrial activity

Sperm mitochondrial activity in vitro was evaluated by staining with MitoTracker Deep Red FM (Thermo Fisher Scientific Inc., Waltham, MA) as described previously [28]. Epididymal sperms from five male mice were incubated in the medium containing 500 nM MitoTracker Deep Red FM for 30 min at 37 °C and observed using a confocal laser microscope (LSM 510 Meta: excitation at 633 nm and emission at 650 nm).

### Evaluation of ROS and reactive nitrogen species (RNS) in sperm

The sperm (2.4 × 10^5^/mouse) was collected from five male mice and subjected to detection of ROS and RNS. ROS and RNS were detected by staining with two fluorescent dyes in the ROS-ID® ROS/RNS detection kit (ENZ-51001-200, Enzo, Farmingdale, NY). The fluorescent sperm were used for estimation of the percentage of stained population. The fluorescence intensities were quantified by using the BZ-analyzer software (Keyence Japan, Osaka, Japan). In each experiment, at least 200 sperm were counted in randomly selected microscopic fields and repeatedly assessed in quintuplicate experiments.

### Statistical analysis

Significant differences (*p* values) between two groups were calculated by using a Student’s *t* test. Differences among three or more groups were evaluated using one-way analysis of variance. In the current study, *p* value below 0.05 was considered significant. Results are expressed as the mean ± SE.

## Results

### Restricted expression of eCS

**Fig. 2:**
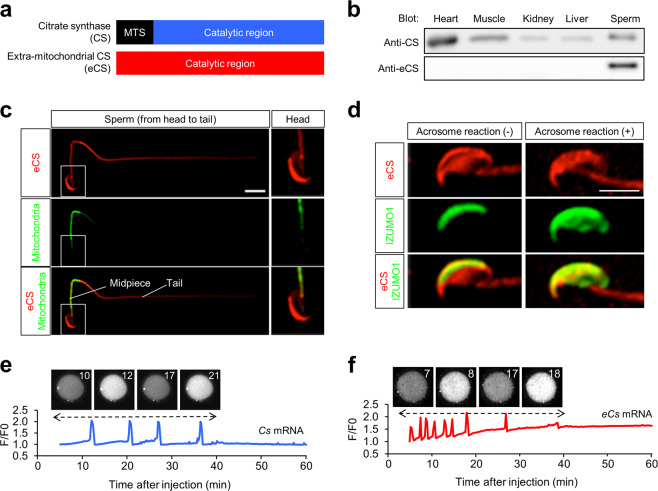
Localization of eCS in the sperm and its involvement in egg activation. **a** Comparison of amino acid (aa) sequences of mouse CS (NP_080720, 464 aa) and eCS (AAH50750, 466 aa) obtained from the NCBI database. MTS mitochondrial-targeting sequence. **b** Abundance of CS and eCS in various tissues. Protein extracts were resolved by electrophoresis and visualized using anti-CS and anti-eCS polyclonal antibodies (polyAbs). Heart, muscle, kidney, liver, sperm. **c** Subcellular localization of eCS in the sperm. The sperm were immunostained with an anti-eCS polyAb (red) and stained with MitoTracker Green (green). Scale bar, 10 μm. **d** Comparison of the expression patterns of eCS before and after the acrosome reaction. The sperm were immunostained with anti-sCS polyAb (red) and anti-IZUMO1 monoclonal Ab (green). Scale bar, 10 μm. The pattern of calcium ion (Ca^2+^) oscillation in eggs injected with Oregon green after the injection of *Cs* (**e**) or *eCs* mRNA (**f**). *Cs* and *eCs* mRNA (2–3 pg/egg) were injected into the eggs at 0 min, and the fluorescent images were then acquired every 10 s. Total 8 and 27 eggs were examined in quintuplicate experiments. The ratio (*F*/*F*_0_) of the fluorescence intensity before the injection (*F*_0_) to that after the injection (*F*) is shown. A dotted line with a double-ended arrow indicates the period of Ca^2+^ oscillation. **e**, **f** Images in the top left show Ca^2+^ oscillations in the eggs and time (s) after the injection of *Cs* or *eCs* mRNA.

CS and eCS proteins (GenBank accession no. NP_080720.1 and NP_082221.2) share high similarity (91.4%) and harbor a conserved mitochondrial retention signal (Fig. 2a and Supplementary Fig. 1). However, eCS lacks the mitochondrial-targeting sequence (MTS), which is important for mitochondrial localization (Fig. 2a). An eCS-specific sequence is located between amino acids 216 and 229 (IYRNLYREDRNIEA), and this was used for the production of an eCS-specific polyAb (Supplementary Fig. 1). In all organs examined, *Cs* mRNA was expressed, but the expression of *eCs* mRNA was limited to the testis (Supplementary Fig. 2a). Moreover, eCS was only detected in the epididymal sperm (Fig. 2b and Supplementary Fig. 2b). We used two Abs, an anti-CS polyAb that recognizes both CS and eCS, and an anti-eCS (eCS-specific) polyAb. The molecular weight of CS was estimated to be 44 kDa, smaller than the detected protein in the sperm extract (46 kDa), which was consistent with that of eCS. From this result, we considered that both CS and eCS would be expressed in the sperm, but their bands were overlapped because their sizes were found to be the same.

We next examined the subcellular localization of CS and eCS. The sperm were immunostained with anti-CS and anti-eCS polyAbs, and counterstained with MitoTracker Green FM (Fig. 2c and Supplementary Fig. 3). CS functions in the mitochondria [29]; in the sperm, the mitochondria are packed in the midpiece [30]. We observed that eCS was distributed in the sperm head and tail, and also in the midpiece (Fig. 2c), whereas CS was localized mainly in the tail and midpiece (Supplementary Fig. 3). Since the eCS signal was intense in the sperm head, we hypothesized that it might function at this location.

### eCS localization in the sperm head

Before sperm-egg fusion, the plasma membrane and the outer acrosomal membrane are removed from the sperm head, in a process termed the AR [31]. Following the release of the acrosomal components sp56 [32], MC101 [33], and acrin1 [34] from the head, the sperm factor must be retained in the sperm head. Hence, we examined the subcellular localization of eCS in the sperm head before and after the AR. We assessed the acrosomal status by using peanut agglutinin lectin [35], which specifically binds the outer acrosomal membrane. As shown in Supplementary Fig. 4, eCS was localized and remained in the sperm head after the AR. We then used an anti-IZUMO1 mAb to detect IZUMO1, an integral membrane protein of the sperm, essential for the sperm-egg fusion [36]. Because IZUMO1 is present in the inner acrosomal region before the AR, the anti-IZUMO1 mAb reacts with the sperm head only after permeabilization. Immunostaining of the sperm with both anti-eCS polyAb and anti-IZUMO1 mAb revealed that eCS remained in sperm head after the AR (Fig. 2d). This observation suggests that eCS is localized in the inner region of the sperm head.

### *Cs* and *eCs* transcripts induce Ca^2+^ level oscillation in the egg

The mouse sperm triggers Ca^2+^ level oscillation in the egg during IVF [37]. Because Ca^2+^ oscillation is observed only after the sperm-egg fusion, the sperm fused with the egg introduces a sperm-derived factor into the egg to induce the oscillation. We microinjected mouse sperm extracts (0.2 pg/egg) into an egg to observe Ca^2+^ oscillation in the egg (Fig. 1b, c). To examine the ability of CS and eCS to induce Ca^2+^ oscillation in the egg, we injected in vitro-synthesized *Cs* or *eCs* mRNA into MII-arrested eggs. The amplitude of Ca^2+^ oscillation induced by both mRNAs was comparable with that observed for mouse sperm extracts (Fig. 2e, f). Although Ca^2+^ oscillations continue in egg injected mouse sperm extracts during 60 min, *Cs* or *eCs* mRNA injection only induces Ca^2+^ oscillations of egg lasting early period (30–40 min). Based on this observation, we concluded that both CS and eCS are able to initiate Ca^2+^ oscillation in the egg, but their abilities are imperfect and limited to the initial period of Ca^2+^ oscillation.

### eCS contributes to male fertility by affecting first spike of Ca^2+^ oscillation

**Fig. 3:**
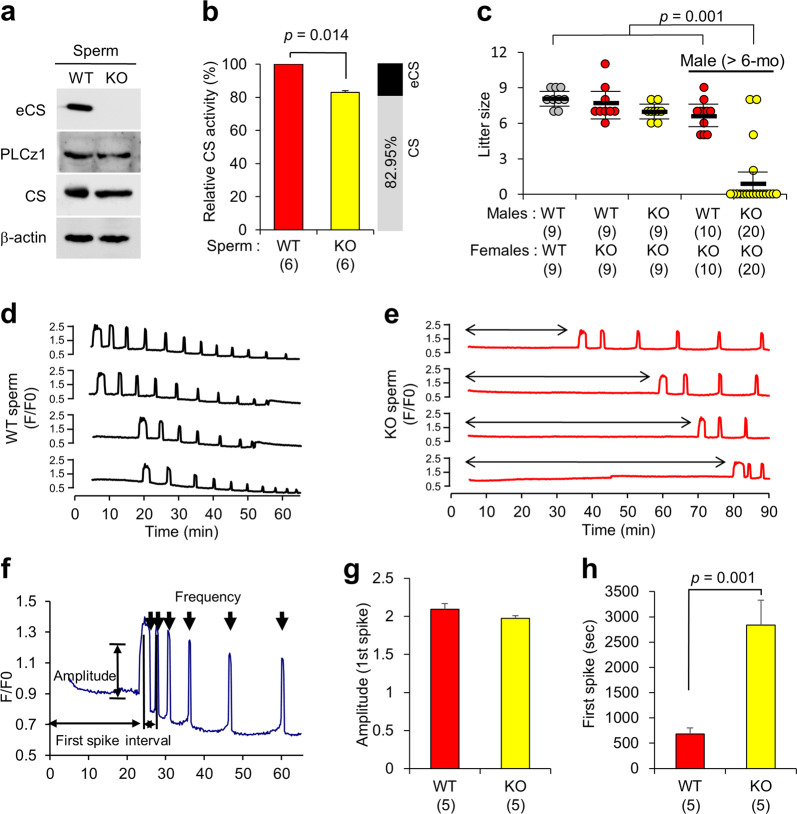
Characterization of the *eCs*-KO sperm. **a** Immunoblotting of testis protein extracts. Blots were probed using antibodies against sperm proteins, as indicated. WT wild-type; KO *eCs*-KO; PLCz1 phospholipase C zeta 1. **b** Citrate synthase activity in the sperm. The cauda epididymal sperm from WT and *eCs*-KO mice were homogenized and proteins were extracted; enzyme activity was measured using acetyl-coenzyme A and oxaloacetate as substrates. Gray and black portions of the bar indicate the ratio of citrate synthase activity attributed to CS and eCS, accordingly. Total numbers of males examined are indicated in parentheses. **c** Fertility of WT and *eCs*-KO males. Male mice were mated with WT and *eCs*-KO females. In each group, nine males were tested. Older WT and *eCs*-KO male mice (>6-month old) were mated with *eCs*-KO females (8–10-week old). In total, 10 and 20 older males were tested, respectively. **d**, **e** Ca^2+^ oscillation in the zona pellucida (ZP)-intact eggs after incubation with WT or *eCs*-KO sperm. For the experiment, 28 and 30 eggs were examined. A line with a double-ended arrow indicates a delayed first spike of Ca^2+^ oscillation in eggs following incubation with the *eCs*-KO sperm. **f** Representative Ca^2+^ oscillation. The amplitude, first spike, interval, and frequency during Ca^2+^ oscillation were quantified. Amplitude (**g**) and first spike (**h**) were compared for eggs fused with WT and *eCs-*KO sperm. Data (**g**, **h**) were calculated based on Ca^2+^ oscillation in 27 and 8 eggs in response to WT and *eCs*-KO sperm. The numbers in parentheses indicate those of the males examined. Values are expressed as the mean ± SE.

To examine the in vivo role of eCS, we generated e*Cs-*KO mice in the C57BL/6NTac background by homologous recombination in embryonic stem cells. The *eCs-*KO mice were born healthy and yielded morphologically normal sperm (Supplementary Fig. 5). The motility of *eCs*-KO and WT sperm was comparable, as determined by examining various parameters (Supplementary Fig. 6). In addition, PLCz1 was detected in both *eCs*-KO and WT sperm (Fig. 3a and Supplementary Fig. 7). PLCz1 was localized in the postacrosomal region of the sperm head, and we did not note any differences in the protein’s localization pattern in the *eCs*-KO and WT sperm (Supplementary Fig. 8). However, the CS activity (total CS activity including eCS) was significantly reduced in the *eCs*-KO sperm (Fig. 3b; the CS/eCS activity ratios 82.9 ± 4.6% and 17.1 ± 4.6% in CS and eCS activity, respectively), indicating that the enzymatic activity of eCS was similar to that of CS. In IVF, two-cell embryos were formed from the *eCs*-KO sperm collected from 8–12-week-old males (Supplementary Fig. 9a). However, *eCs*-KO sperm collected from 6-month-old males exhibited significantly decreased two-cell formation, compared with WT sperm collected from 6-month-old males (Supplementary Fig. 9a). Similar to IVF results, *eCs*-KO males were fertile, but their litter size was remarkably reduced in 6-month-old males (Fig. 3c). Based on these observations, we hypothesized that eCS could be involved in male reproduction, with its contribution to male fertility increasing with age, conceivably because of reduced mitochondrial CS activity.

We next examined the pattern of Ca^2+^ oscillation in WT eggs fused with *eCs*-KO sperm (Fig. 3d, e), by continuously measuring Ca^2+^ concentration during incubation with the sperm. Ca^2+^ oscillation occurred in eggs fused with WT sperm, and was also induced in eggs fused with *eCs*-KO sperm. However, in the latter group, the oscillation pattern was disturbed; specifically, the emergence of the first spike was noticeably delayed (Fig. 3e). Interestingly, no first spike during 90 min occurred in 22 of 30 eggs (73.3%) after the insemination with *eCs*-KO sperm (Supplementary Fig. 10). We next assessed the pattern of Ca^2+^ oscillation in eggs fused with *eCs*-KO sperm in terms of amplitude, interval, frequency, and the first spike of Ca^2+^ oscillations (Fig. 3f), and compared these parameters with those in eggs fused with WT sperm (Fig. 3g, h, and Supplementary Fig. 9b, c). Although no significant differences were observed in the amplitude and interval (Fig. 3g and Supplementary Fig. 9b), the frequency of Ca^2+^ oscillation was lower in eggs fused with *eCs*-KO sperm (4.0 ± 0.4 by 90 min) than in eggs fused with WT sperm (7.4 ± 0.7 by 90 min) (Supplementary Fig. 9c). The initiation of the first spike was strongly delayed in eggs fused with *eCs*-KO sperm (2838.8 ± 489.2 s) compared with that in eggs fused with WT sperm (680.5 ± 121.0 s; Fig. 3h). Of note, in the above experiments, time 0 min was the start time of sperm incubation with eggs, not the time of initiation of the sperm-egg fusion. Hence, if there was no retardation of the sperm-egg fusion, the above observations would indicate that eCS participates in the first spike of Ca^2+^ oscillation.

### Metabolomic analysis of the *eCs*-KO sperm

Since the *eCs*-KO mouse exhibits age-dependent male infertility (Fig. 3c), we hypothesized that the metabolism of the *eCs*-KO sperm could be slowed with age. To investigate this possibility, we quantified the metabolites in the sperm by using GC–MS. We compared the metabolomic profiles of the *eCs*-KO sperm collected from 3-month-old and 6-month-old mice with those of WT sperm collected from 3-month-old and 6-month-old mice.

**Fig. 4:**
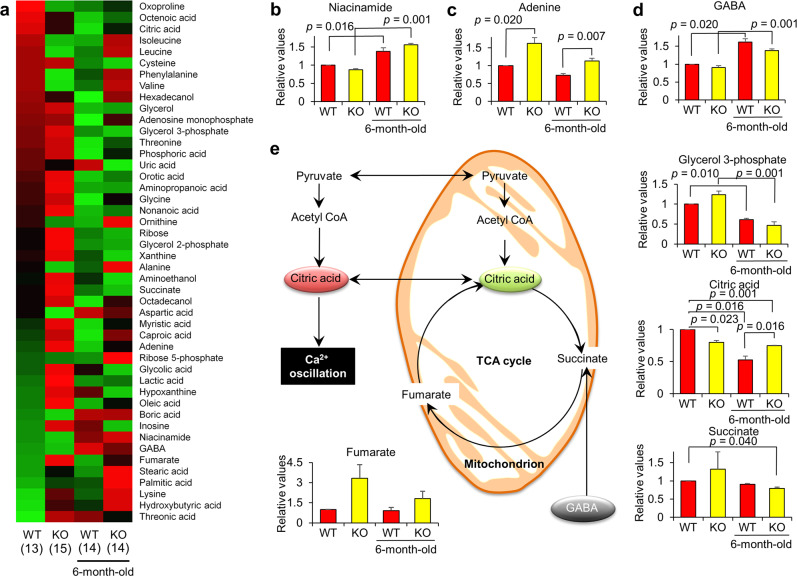
Metabolome analysis of WT and *eCs*-KO sperm. **a** Heatmap of metabolites present in different quantities in the sperm. Profiles of sperm metabolites were ordered from high expression (red) to low expression (green), based on metabolic level of WT sperm collected from 3-month old. Heatmap represents profiles of WT and *eCs*-KO sperm metabolites, collected from 3- and 6-month-old mice, respectively. **b**, **c**, **d** TCA cycle-related metabolites. Niacinamide (**b**), Adenine (**c**), and GABA (**d**). **e** Pathway diagram of TCA cycle and multiple intermediates. The graphs show the relative values of metabolite quantities in WT and *eCs*-KO sperm, calculated in relation to the levels in WT sperm. The value in WT sperm (the leftmost bar) was arbitrarily set at 1.0. Prepared sperm from 2–3 male mice were examined for each sample, and six independent experiments were repeated per group. Total numbers of males examined are indicated in parentheses. Values are expressed as the mean ± SE.

The GC–MS approach allowed us to identify and quantify 46 metabolites (displayed in a heatmap in Fig. 4a). The amounts of these metabolites in *eCs*-KO sperm collected from 3-month-old mice were noticably different from those in the WT sperm collected from 3-month-old mice. Specifically, the levels of 26 metabolites were increased and those of eight metabolites were reduced in the *eCs*-KO sperm. Otherwise, the levels of most metabolites were reduced in the WT sperm collected from 6-month-old mice compared with those of WT sperm collected from 3-month-old mice, while some of them were elevated in the *eCs*-KO sperm from 3-month-old mice. To explain these alterations, we focused on seven metabolites [niacinamide, adenine, 4-aminobutyric acid (GABA), glycerol 3-phosphate, citrate, succinate, and fumarate].

Niacinamide (also known as nicotinamide) is a precursor of nicotinamide adenine dinucleotide as a precursor of adenosine triphosphate (ATP) [38]. Niacinamide levels significantly increased with age in both *eCs*-KO and WT sperm (Fig. 4b). By contrast, adenine levels were significantly increased in the *eCs*-KO sperm (Fig. 4c), implying that a combination of eCS deficiency and aging enhanced nicotinamide adenine dinucleotide production.

GABA is an inhibitory neurotransmitter in the central nervous system. Intracellular GABA enhances the tricarboxylic acid (TCA) cycle, because succinate semialdehyde is a metabolite of GABA, which leads to succinate production [39]. GABA levels significantly increased with age in both *eCs*-KO and WT sperm (Fig. 4d). Conversely, the levels of glycerol 3-phosphate, a glycolysis intermediate, were reduced with age in both *eCs*-KO and WT sperm (Fig. 4e).

Citrate levels were significantly decreased in the *eCs*-KO sperm from 3-month-old mice (Fig. 3b), but were maintained in the *eCs*-KO sperm from 6-month-old mice (Fig. 4e). Fumarate levels tended to increase in the *eCs*-KO sperm, while succinate was maintained at the WT sperm level. These observations indicated that eCS deficiency in combination with aging enhances the TCA cycle, presumably leading to the shortage of extra-mitochondrial citrate and sperm dysfunction when inducing Ca^2+^ oscillation in the egg after fusion with the sperm.

To next explore the effect of oxidative stress on sperm dysfunction with age, we estimated ROS and RNS levels between WT and *eCs*-KO sperm. Although both ROS and RNS levels in WT sperm were significantly increased with age (Supplementary Fig. 12b, c), there were no significant differences in both levels between WT and *eCs*-KO sperm (Supplementary Fig. 12b, c).

## Discussion

In the present study, we showed that eCS is a novel sperm factor that induces Ca^2+^ oscillation in the mouse egg. Further, Ca^2+^ oscillation of *eCs*-KO sperm demonstrates that eCS plays an important role in initiation of first spike, although they are fertile (Fig. 3c–h). Intriguingly, eCS is mainly localized in acrosome of sperm head, differ with that of CS (Fig. 2). Also, eCS has a CS activity (Fig. 3b), implying independent role of eCS in sperm. Notably, eCS is likely involved in synthase of citrate relating to sperm function in the extra-mitochondrial cytoplasm (Fig. 4). Moreover, eCS expression in WT sperm was decreased age dependently (Supplementary Fig. 11a, c). These data may explain the reason why *eCs*-KO sperm show age-dependent decrease in fertility.

Injection of *eCs* transcripts induced Ca^2+^ oscillation in the egg (Fig. 2f), and the initiation of Ca^2+^ oscillation was strongly delayed in egg fused with *eCs*-KO sperm (Fig. 3 and Supplementary Fig. 10). Previous reports have strongly supported the notion that PLCz1 is a sole molecule responsible for Ca^2+^ oscillation [9, 40]. Specifically, the pattern of Ca^2+^ oscillation in mouse eggs injected cRNA mouse sperm PLCz is quite similar to that in an egg fused with a sperm, and the removal of PLCz1 from sperm extracts results in failure to induce Ca^2+^ oscillation [9]. As described recently [10, 11], sperm derived from a *Plcz1*-KO male failed to trigger Ca^2+^ oscillation in the egg and caused polyspermy in vitro. However, *Plcz1*-KO male mice are subfertile and sustained minimum fertility. These studies suggest that PLCz1 does not act alone to initiation of egg activation, at least in mouse.

In the current study, we demonstrate that eCS deficiency leads to a substantial delay in the first spike of Ca^2+^ oscillation in the egg. According to our data, it is likely that the enzymatic activity of CS triggers Ca^2+^ oscillation. Because eCS synthesizes citrate, as demonstrated by the observation that citrate levels were significantly reduced in the *eCs*-KO sperm compared with WT sperm, while the mitochondrial CS accounted for the majority of CS activity in the whole-sperm extract (Fig. 3b). We expect that the contribution of eCS to enzymatic activity of citrate synthesis is low in whole sperm, but becomes substantial in the sperm head because of the high abundance of eCS therein (Fig. 2c, d). Since citrate diffuses throughout the sperm cytoplasm [41], citrate synthesized by CS presumably affects the citrate-mediated sperm functions. As shown in Fig. 2e, CS triggers the first Ca^2+^ rise, implying that CS and CS-synthesized citrate may function as a substitute of eCS. In general, a global decrease in mitochondrial activity occurs with age [42]. Accordingly, male fertility was reduced in *eCs*-KO mice with age (Fig. 3c). This indicates that decreased CS function with age may lead to male fertility decline.

**Fig. 5:**
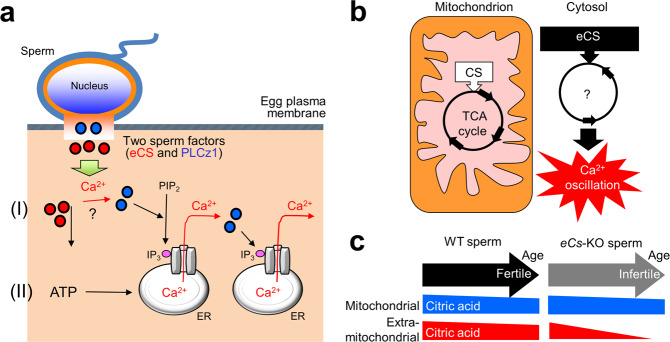
Schematic model of the role of eCS in Ca^2+^ oscillation in the eggs. **a** Following sperm fusion with the egg, the sperm-derived factors trigger Ca^2+^ oscillation in the egg. Two sperm-derived factors, phospholipase C zeta 1 (PLCz1) and eCS, are involved in Ca^2+^ oscillation in the mouse egg. Red circles, eCS; blue circles, PLCz1. (I) eCS may function to initiate Ca^2+^ oscillation, especially the first spike, alone and/or assisting PLCz1 to induce Ca^2+^ oscillation. (II) eCS probably produces ATP in the extra-mitochondrial space, which participates in the initiation of Ca^2+^ oscillation. **b** CS and eCS in the mammalian cell. **c** Relationship between sperm metabolic patterns and male fertility. With aging, the citrate content in the extra-mitochondrial space in the sperm is reduced. Such reduction in citrate levels may result in reduced male fertility.

The data presented herein suggest the possibility that at least two sperm factors, eCS and PLCz1, are involved in Ca^2+^ oscillation in the mouse egg [(I) in Fig. 5a]. This hypothesis is supported by an earlier report [43] that meiotic resumption can be induced by the injection of a cytosolic sperm fraction lacking sperm-borne oocyte-activating factors, including PLCz1, suggesting the existence of an additional sperm factor.

Although both *eCs* and *Plcz1*-KO sperm elicit delayed triggering of Ca^2+^ oscillation, they exhibit completely different patterns of Ca^2+^ oscillation. Even though the first spike was delayed in an egg inseminated with *eCs-* and *Plcz1*-KO sperm, a relatively regular oscillation occurred after the initiation of Ca^2+^ oscillation in the egg inseminated with *eCs*-KO sperm (Fig. 3e). By contrast, few frequencies of Ca^2+^ oscillation are observed in an egg inseminated with *Plcz1*-KO sperm, and lasting a short time [11]. Hence, eCS might independently control the early step of Ca^2+^ oscillation (i.e., initiation; first spike), whereas PLCz1 could continue to regulate the latter step of Ca^2+^ oscillation (i.e., persistence).

Even though *eCs*-KO sperm induced insufficient Ca^2+^ oscillation, the *eCs*-KO males were fully fertile, but the period of fertility was relatively shortened. The age-dependent reduction of litter size may be related to the ATP production in the egg. In human granulosa–luteal cells, ATP evokes Ca^2+^ oscillation in the absence of extracellular Ca^2+^, while sustained Ca^2+^ oscillation requires extracellular Ca^2+^ [44]. In mouse egg, the initial ATP increase occurs with the first Ca^2+^ rise, and the second ATP increase takes place 1 h later [45]. In other words, the first Ca^2+^ rise depends on ATP production. By contrast, the second and additional increases of the Ca^2+^ levels are independent of ATP production but depend on the extracellular Ca^2+^ concentration. Based on previous reports and the findings of current study, we propose that eCS-driven ATP production triggers the first Ca^2+^ rise that corresponds to Ca^2+^ release from intracellular storage; the ATP demand then becomes lower, presumably shifting the mechanism of increasing Ca^2+^ levels from dependence on intracellular Ca^2+^ to dependence on extracellular Ca^2+^ [(II) in Fig. 5a]. Hence, since aging leads to mitochondrial dysfunction, the decrease of male fertility conceivably accounts for the reduced CS activity with aging. Actually, aging leads to a significant decrease in CS expression in the WT sperm (Supplementary Fig. 11a, b), but there was no significant difference in mitochondrial activity between WT and *eCs*-KO sperm (Supplementary Fig. 12a).

On the other hand, ROS and RNS are mainly produced in mitochondria [46] and mitochondria dysfunction causes their excess production and subsequent abnormal energy metabolism. Mitochondrial dysfunction also causes severe oxidative stress with age in both eggs and sperm [47]. Therefore, oxidative stress is conceivably committed to mitochondrial dysfunction of *eCs*-KO sperm with age. Unexpectedly, the effect of oxidative stress was unseen between WT and *eCs*-KO sperm (Supplementary Fig. 12b, c), implying that the decrease of *eCs*-KO male fertility with age may be unrelated to oxidative stress.

In the current study, metabolomic analysis of the WT and *eCs*-KO sperm revealed that the citrate levels were significantly lower in *eCs*-KO sperm than those in WT sperm, with the fumarate levels showing a significant increase in the *eCs*-KO sperm compared with the WT sperm (Fig. 4b). Alterations of metabolites related to the TCA cycle and energy metabolism in the *eCs*-KO sperm suggested a deficiency in ATP production caused by dysregulation of the TCA cycle by citrate deficiency. Because the majority of cellular energy is produced by the electron transport chain in a process of ATP generation in the mitochondria [48], decreased levels of citrate in the TCA cycle suggest a deficiency of ATP production in the *eCs*-KO sperm. However, eCS does not contain MTS and is mainly localized in the acrosome in the sperm head (Fig. 2a, c), not the tail, where the mitochondria reside. These discrepancies support the possibility that eCS may function as a sole CS related to the TCA cycle in the extra-mitochondrial space (Fig. 5b).

According to metabolomic analysis of cancer cells, citrate in tumor tissues of colon and stomach was lower than that of normal tissues [49]. In addition, three TCA metabolites succinate, fumarate, and malate were significantly higher in tumor of colon and stomach tissue [49]. Interestingly, metabolic profiles of the *eCs*-KO sperm were quite similar to those of both colon and stomach cancers, implying that the fertility decline observed in the *eCs*-KO mouse with age may arise from the enhancement of a TCA cycle-centric metabolism.

The expression of an alternatively spliced form of *Cs* that lacks the mitochondrial retention signal (eCS) is restricted in human tissues [50], and the conserved certain parts of the CS protein identified by Karpusas et al. [51] can function as extra-mitochondrial enzymes. This implies that the role of an eCS might be broadly conserved during different biological events in mammals (Fig. 4b).

In this study, we showed the role of eCS in age-related changes in male fertility. Importantly, we demonstrated an abnormal metabolism in the *eCs*-KO sperm with age, resulting from CS deficiency in the TCA cycle. The changes in citrate levels in *eCs*-KO sperm may reflect the inactivity of the TCA cycle and could be used as markers to discriminate the aging infertile male from aging fertile male (Fig. 5c).

In the present study, we showed that initiation of Ca^2+^ oscillation was significantly delayed in eggs fused with *eCs*-KO sperm despite the normal expression of *Plcz1*. This result offers a novel insight into the role of sperm factor, because *eCs*-KO males were fertile initially. Aberrant function of *Plcz* is able to explain egg activation failure in infertile patients [52]. Otherwise, eCS dysfunction in the sperm may explain age-dependent male infertility in the presence of PLCZ1. *eCs*-KO male mice are useful for analyzing unexplained male infertility with age, presumably contributing to development of diagnostic methods in patients with age-dependent male infertility.

## Supplementary information

Revised supplementary Materials
