# Supplementary material for: Extra-mitochondrial citrate synthase initiates calcium oscillation and suppresses age-dependent sperm dysfunction
Source: Lab Invest. 2019 Dec 19;100(4):583–95. doi: 10.1038/s41374-019-0353-3 (PMC7096335; doi:10.1038/s41374-019-0353-3)
Supplement: Supplementary file 1 — Revised supplementary Materials [file 41374_2019_353_MOESM1_ESM.pdf]

Supplementary Materials

Extra-mitochondrial citrate synthase initiates calcium oscillation and suppresses age-dependent sperm dysfunction

Woojin Kang, Yuichirou Harada, Kenji Yamatoya, Natsuko Kawano, Seiya Kanai, Yoshitaka Miyamoto, Akihiro Nakamura, Mami Miyado, Yoshiki Hayashi, Yoko Kuroki, Hidekazu Saito, Yasuhiro Iwao, Akihiro Umezawa, and Kenji Miyado

Supplemental Fig. 1 to 11

```
CS          MALLTAATRLLGAKNSSCLVLAARHASASSTNLKDVLSNLIPKEQARIKTFKQQHG
eCS         MALLTAAAWFLGTKNPPCLVLAARHASASSTNLKDVLRNLIPKEQARIKTFRKKHG
consensus   *******:  :**:**..******************* *************:::**

CS          KTVVGQITVDMMYGGMRGMKGLVYETSVLDPDEGIRFRGYSIPECQKMLPKAKGGE
eCS         KTVVGQITVDMMYGGMRGMKGLVYETSVLDPDEGIRFRGYSIPECQKLLPKAKGGK
consensus   ***********************************************:*******:

CS          EPLPEGLFWLLVTGQMPTEEQVSWLSREWAKRAALPSHVVTMLDNFPTNLHPMSQL
eCS         EPLPEGLFWLLVTGQMPTEEQVSWLSQEWVKRAALPSHVVTMLDNFPTKLHPMSQL
consensus   **************************:**.*****************:*******

CS          SAAITALNSESNFARAYAEGMNRAKYWELIYEDCMDLIAKLPCVAAKIYRNLYREG
eCS         SAAITVLNNESNFARAYAQGMNRTKYWELTYEDCMDLLAKLPCVAAKIYRNLYRED
consensus   *****.**.*********:****:***** *******:*****************.

CS          SSIGAIDSRLDWSHNFTNMLGYTDPQFTELMRLYLTIHSDHEGGNVSAHTSHLVGS
eCS         RNIEAIDSKLDWSHNFTNMLGYTDPQFTELMRLYLTIHSDHEGGNVSAHTSHLVGS
consensus   .* ****:***********************************************

CS          ALSDPYLSFAAAMNGLAGPLHGLANQEVLVWLTQLQKEVGKDVSDEKLRDYIWNTL
eCS         ALSDPYLSFAAALNGLAGPLHGLANQEVLVWLTQLQKEVGEDASDEKLKNYIWNTL
consensus   ************:***************************:*.*****::******

CS          NSGRVVPGYGHAVLRKTDPRYSCQREFALKHLPKDPMFKLVAQLYKIVPNILLEQG
eCS         NSGRVVPGYGHAVLRKTDPRYSCQREFALKHLPKDPMFKLVGQLYKIVPDILLEQG
consensus   *****************************************.*******:******

CS          KAKNPWPNVDAHSGVLLQYYGMTEMNYYTVLFGVSRALGVLAQLIWSRALGFPLER
eCS         KAKNPWPNVDAHSGVLLQYYGMREMNYYTVLFGVSRALGVLSQLIWSRALGFPLER
consensus   ********************** ******************:**************

CS          PKSMSTDGLMKFVDSK--
eCS         PKSMSTDALMKFVNSESG
consensus   *******.*****:*:
```

Supplemental Fig. 1 Alignment of eCS and CS amino acid sequences. Asterisk indicates conserved amino acids sequence between CS and eCS. Red color of amino acid sequence indicates non-conservative sequence. The star, dots, and colons below the alignment represent degree of conservation in columns between two groups. Rabbit anti-eCS polyAb raised against a peptide corresponding to the label under bar sequence.

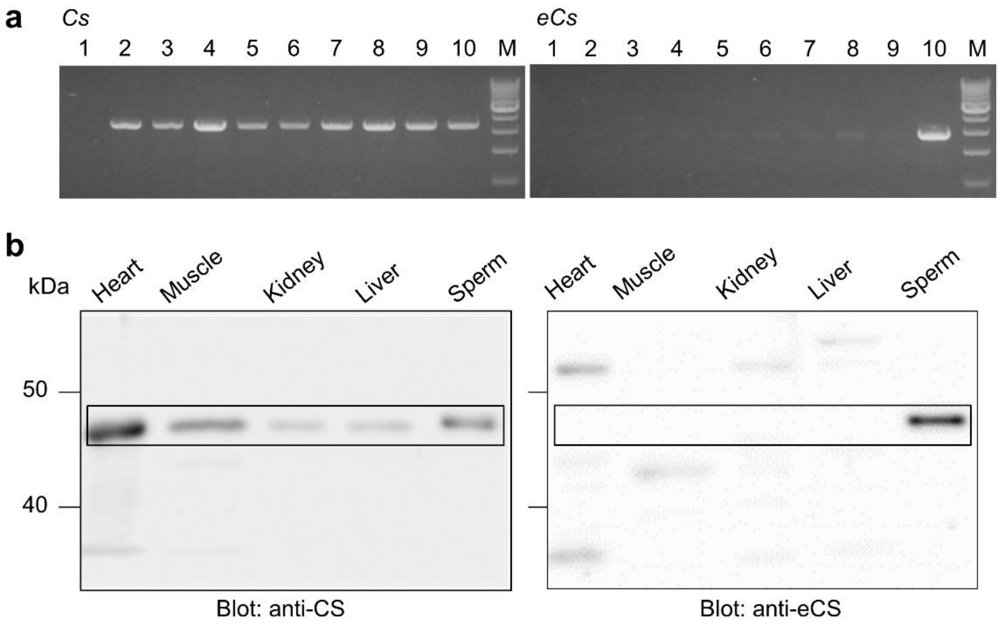

Supplemental Fig. 2 Expression of CS and eCS. **a** Expression of *Cs* and *eCs* transcripts in tissues. mRNA was analyzed by RT-PCR using total RNA from several tissues. 1, negative control (without template); 2, brain; 3, lung, 4: heart; 5, liver; 6, spleen; 7, kidney; 8, muscle; 9, ovary; 10, testis; M, size marker. **b** Immunoblotting for CS and eCS proteins in tissues. Hea, heart; Mus, muscle; Kid, kidney; Liv, liver; Spe, sperm. Black squares indicate bands represented in Fig. 2b.

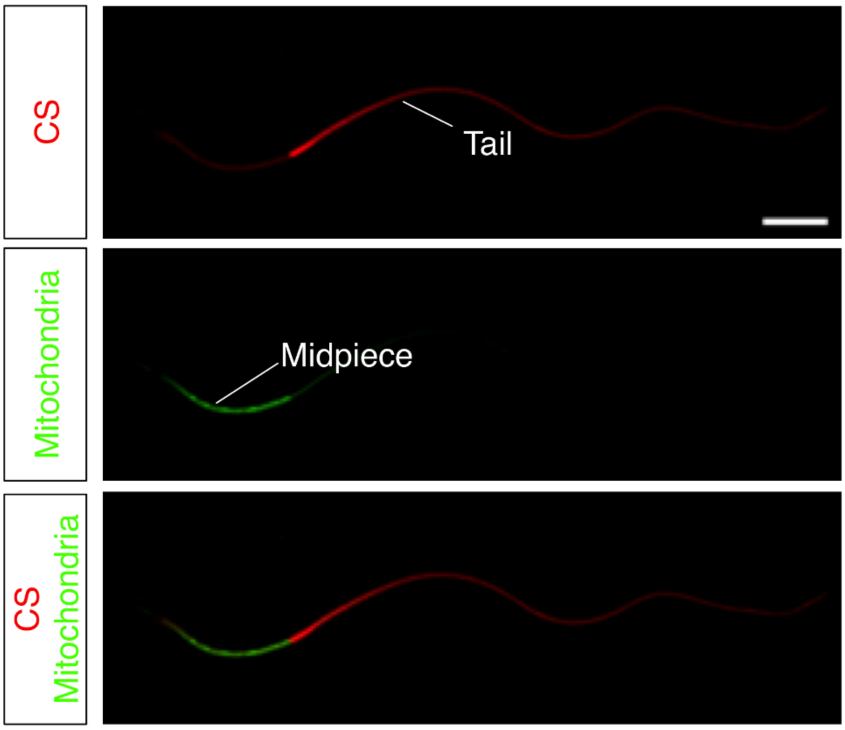

Supplemental Fig. 3 Subcellular localization of CS in the sperm. Sperm were fixed and stained with an anti-CS polyAb (red) and MitoTracker Green FM (green). Scale bar, 10 µm.

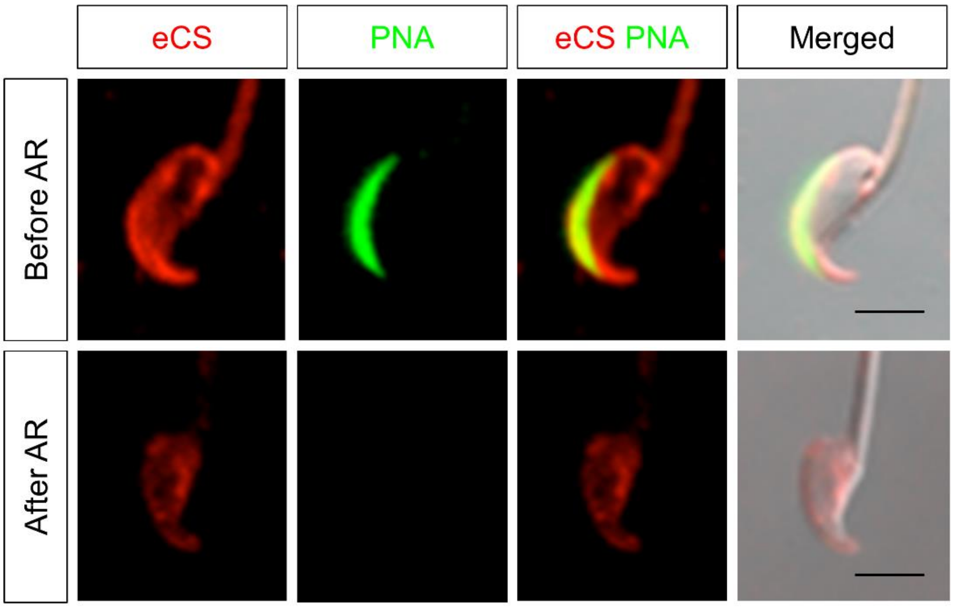

Supplemental Fig. 4 Localization of eCS in the sperm head before and after the acrosome reaction (AR). Sperm were stained with an anti-eCS polyAb (red) and Alexa Fluor 488-conjugated peanut lectin PNA (green). Scale bar, 5 µm.

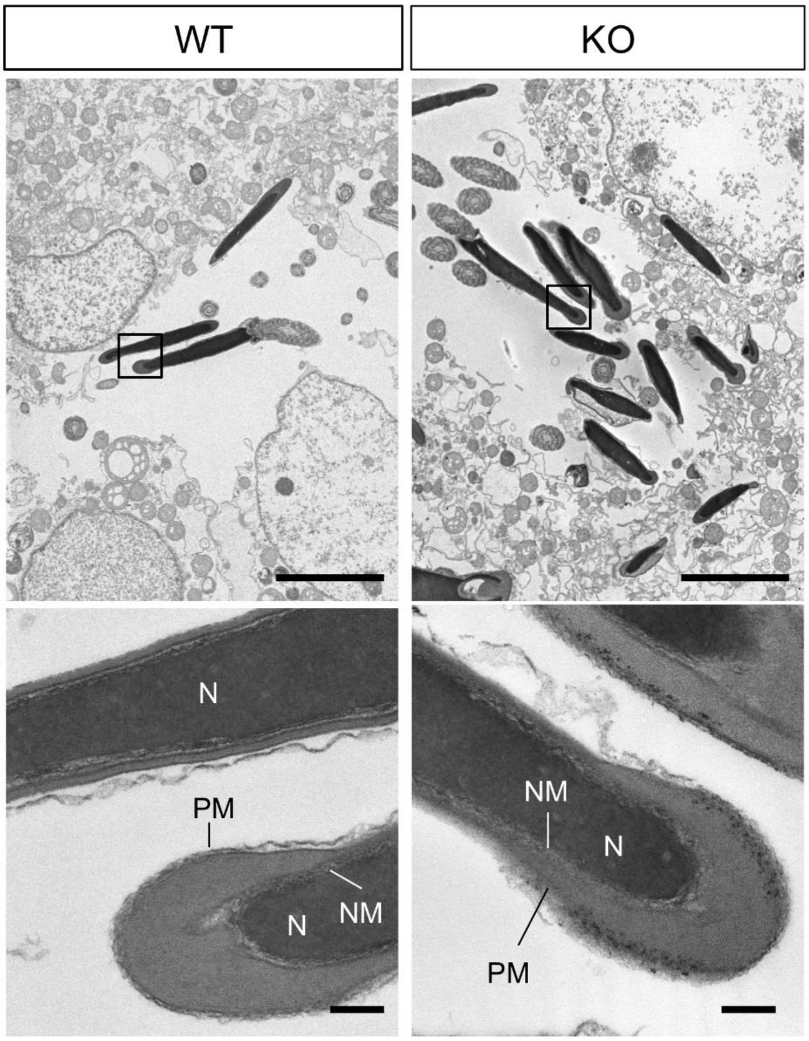

Supplemental Fig. 5 Transmission electron microscopic images. Diagram at left indicates the orientation of the sectioned heads of sperm. (Left) WT, wild-type; (right) KO, *eCs*-KO (upper and lower picture; scale bars: 1 µm and 200 µm). Lower picture shows enlarged view of the black square regions. N, Nucleus; NM, nuclear membrane; PM, plasma membrane.

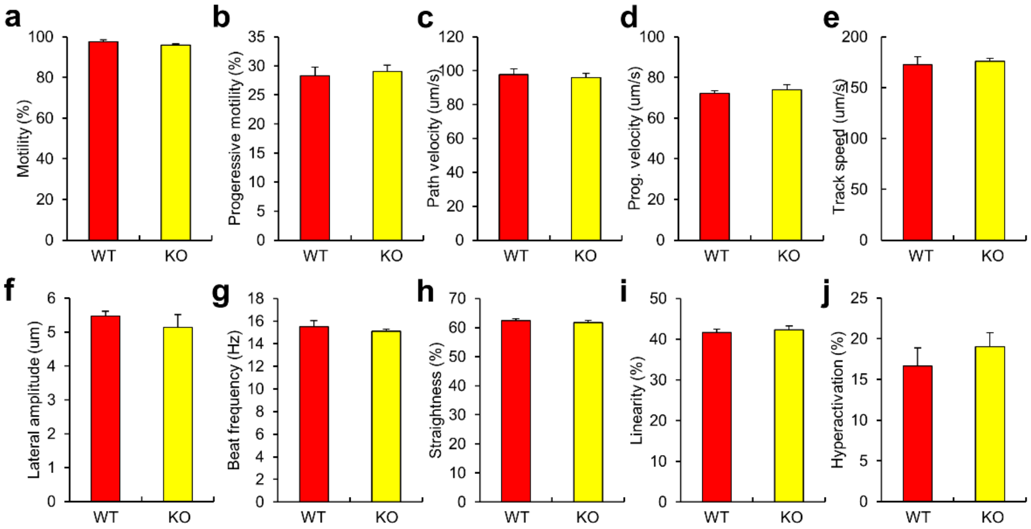

Supplemental Fig. 6 Motility of e*Cs*-KO and WT epididymal sperm. At least 200 sperm were examined from each sperm sample from three different mice. Values represent mean ± SE.

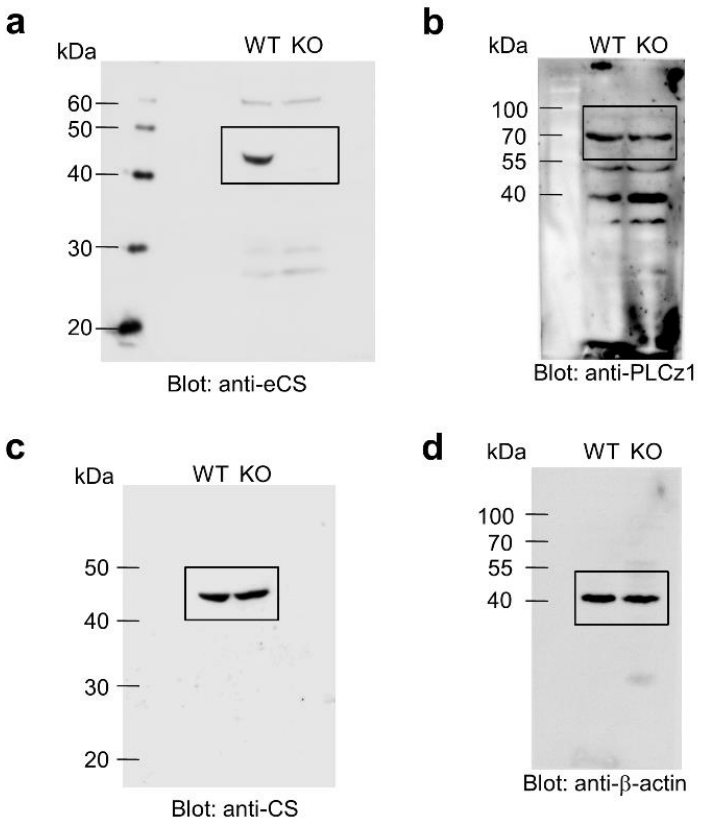

Supplemental Fig. 7 Immunoblotting of epididymal sperm. Black squares indicate bands represented in Fig. 3a.

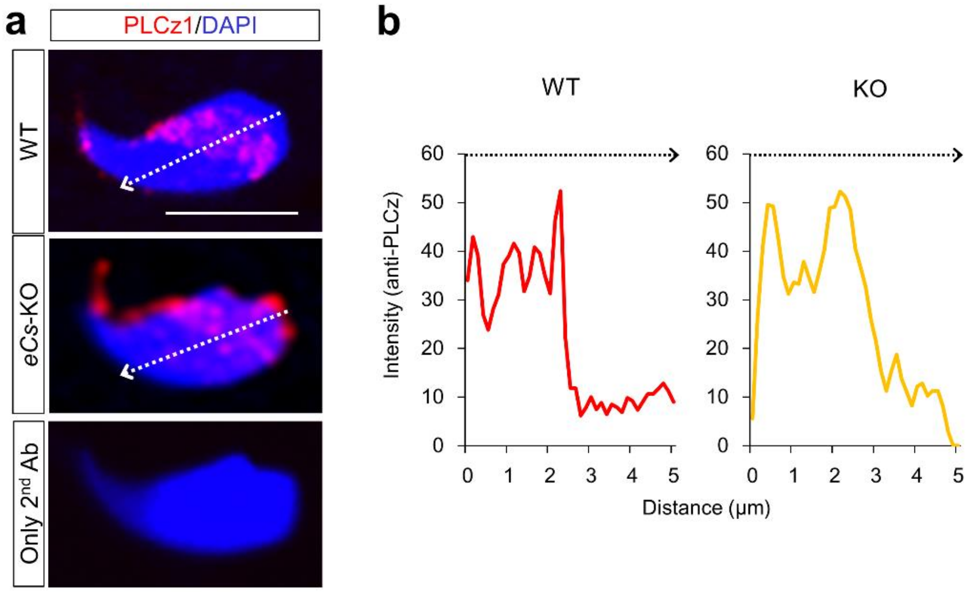

Supplemental Fig. 8 Distribution of PLCz1 in sperm. **a** Immunostaining of PLCz1 in sperm. Sperm were stained with an anti-PLCz1 polyAb (red). Scale bar, 10 μm. **b** The fluorescence intensity profiles of PLCz1-stained areas in sperm head shown in a. Fluorescence intensities were measure along with white dotted lines.

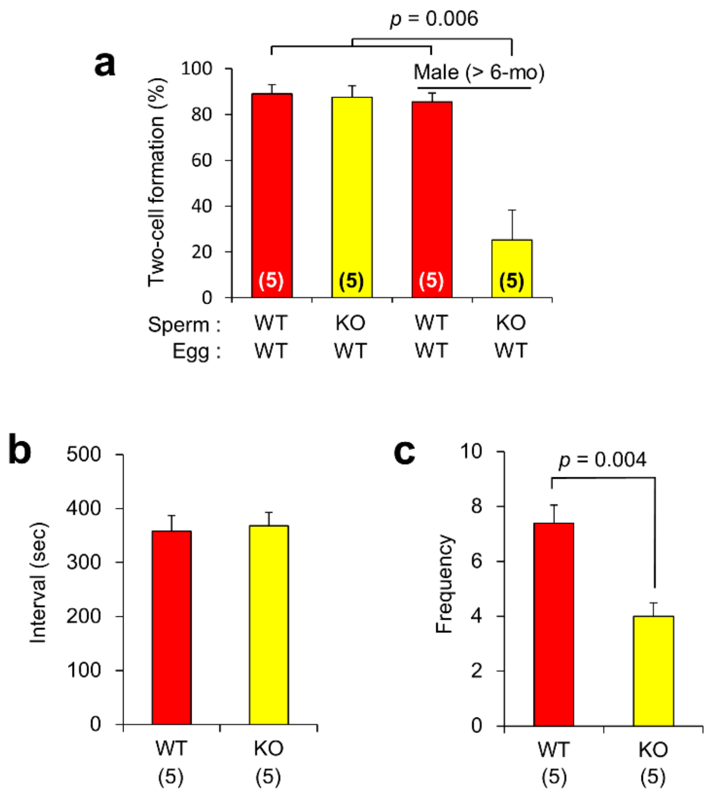

Supplemental Fig. 9 IVF rate and parameters of Ca^{2+} oscillation (interval and frequency) between *eCs*-KO and WT sperm. **a** Rate of egg development to two-cell embryos after *in vitro* fertilization. Capacitated WT, *eCs*-KO, WT (from 6-month-old males), and *eCs*-KO (from 6-month-old males) sperm were incubated for 24 h with cumulus-intact WT eggs. In total, 60, 122, 78, and 68 eggs were examined 24 h after insemination by WT and *eCs*-KO sperm, respectively. The numbers of males examined are indicated in parentheses. **b, c** Parameters of quantification were interval and frequency during Ca^{2+} oscillation. Interval and frequency were compared between eggs fused with WT and *eCs*-KO sperm. Data were calculated based on Ca^{2+} oscillation in eggs in response to WT and *eCs*-KO sperm (26 and 8 eggs). The numbers in parentheses indicate those of the males examined. Values are expressed as mean ± SE.

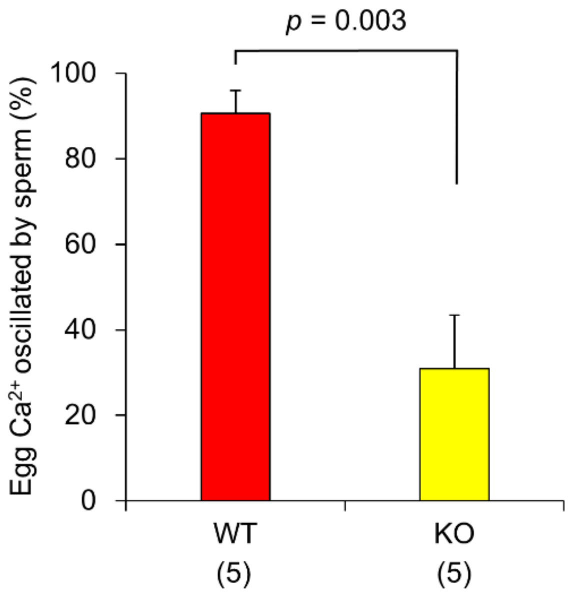

Supplemental Fig. 10 Ca^{2+} oscillation response in eggs after insemination with WT and *eCs*-KO sperm. Total numbers of 28 and 30 eggs were examined after insemination with WT and *eCs*-KO sperm, respectively. The numbers of males examined are indicated in parentheses. Values are expressed as mean ± SE.

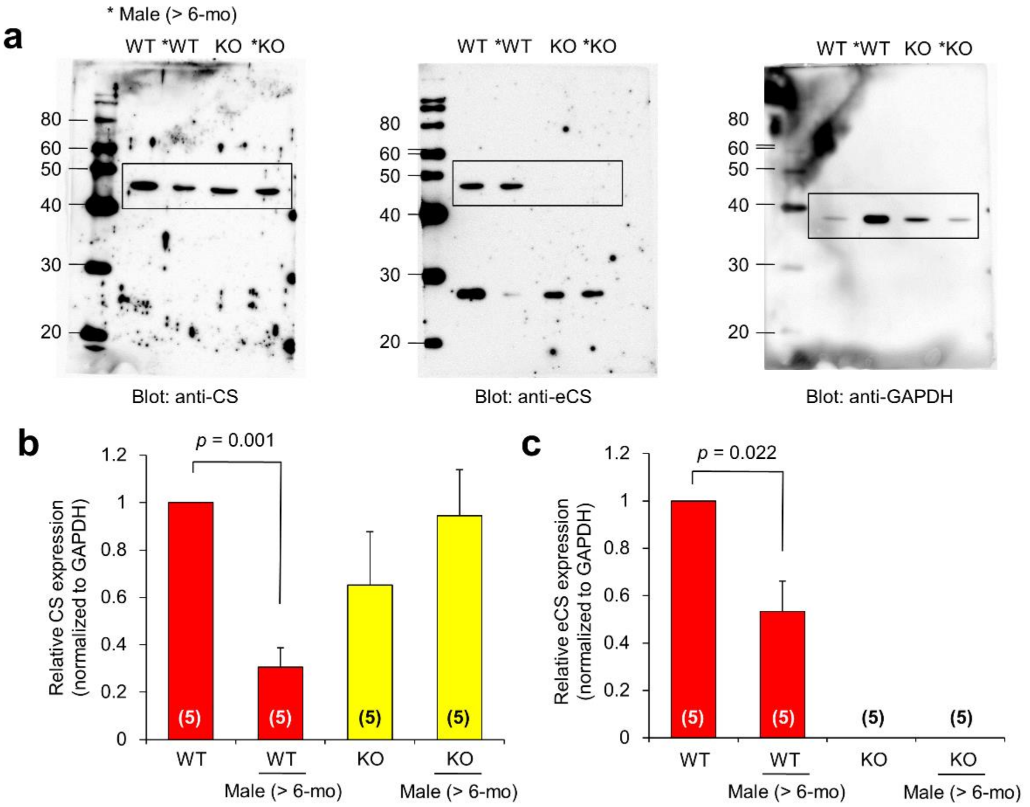

Supplemental Fig. 11 Relative expression of CS and eCS proteins in sperm with age. **a** Representative immunoblots of CS, eCS, and GAPDH in extracts from WT, *eCs*-KO, WT (from 6-month-old males), and *eCs*-KO sperm (from 6-month-old males). GAPDH was examined as a loading control. **b, c** Quantitative comparison of CS and eCS proteins in respective lanes as shown in a. The intensity of bands was measured using ImageJ software. The expression of CS and eCS proteins in each lane was determined by normalizing GAPDH. The band intensity in the WT sperm (the leftmost bar) was arbitrarily set at 1.0. The graph indicates relative expression of CS and eCS proteins in the sperm. The numbers of males examined are indicated in parentheses. Values are expressed as mean ± SE.

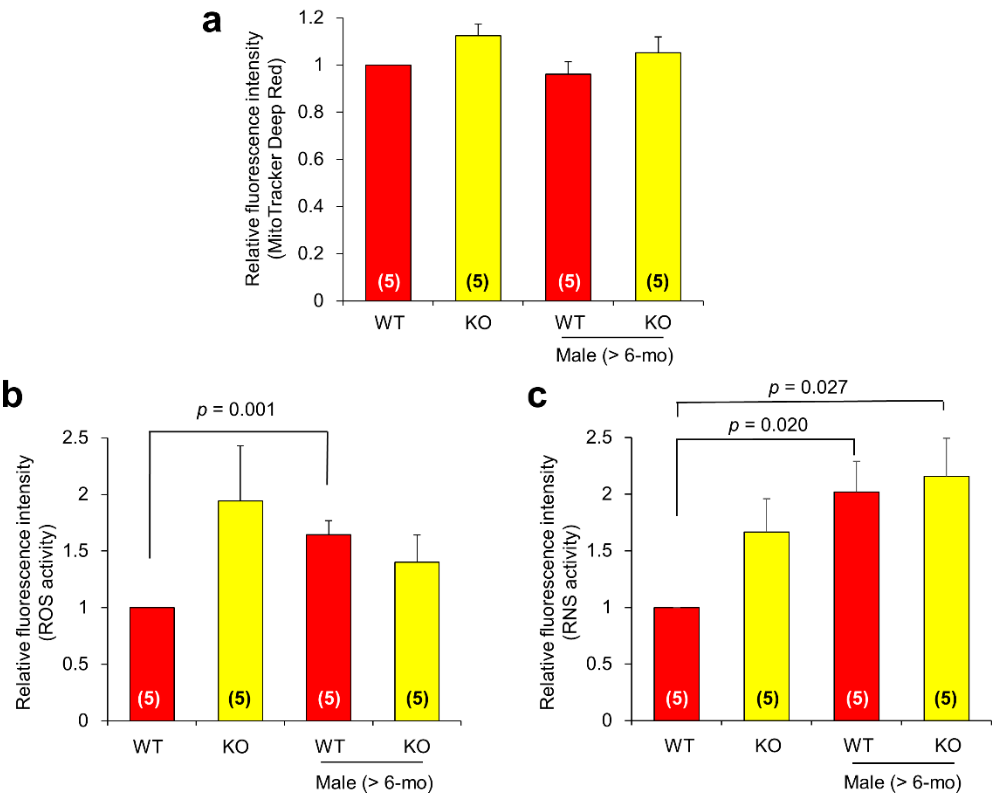

Supplemental Fig. 12 Comparison of mitochondrial activity, and amounts of reactive oxygen species (ROS) and reactive nitrogen species (RNS) between WT and *eCs*-KO sperm with age. **a** Quantification of fluorescence intensities of mitochondrial staining in sperm from WT and *eCs*-KO mice. The signal intensity in WT sperm (the leftmost bar) was arbitrarily set at 1.0. The graph indicates relative signal intensity of mitochondria in the sperm. **b, c** Quantification of fluorescence intensity of ROS and RNS. The signal intensity in WT sperm (the leftmost bar) was arbitrarily set at 1.0. The graph indicates relative intensity of ROS and RNS in the sperm. The numbers of males examined are indicated in parentheses. Values are expressed as mean ± SE.
